# Protopine Protects Mice against LPS-Induced Acute Kidney Injury by Inhibiting Apoptosis and Inflammation via the TLR4 Signaling Pathway

**DOI:** 10.3390/molecules25010015

**Published:** 2019-12-19

**Authors:** Beibei Zhang, Mengnan Zeng, Meng Li, Yuxuan Kan, Benke Li, Ruiqi Xu, Yuanyuan Wu, Shengchao Wang, Xiaoke Zheng, Weisheng Feng

**Affiliations:** 1 College of Pharmacy, Henan University of Chinese Medicine, Zhengzhou 450046, China; zhangs9426@163.com (B.Z.); 17320138484@163.com (M.Z.); limeng31716@163.com (M.L.); kanyx0827@163.com (Y.K.); libk2017@163.com (B.L.); xuruiqi9647@163.com (R.X.); WYY96191711@163.com (Y.W.); wangsc1204@163.com (S.W.); 2 Collaborative Innovation Center for Respiratory Disease Diagnosis and Treatment & Chinese Medicine Development of Henan Province, Zhengzhou 450046, China

**Keywords:** protopine, acute kidney injury, apoptosis, inflammation, TLR4 signaling pathway

## Abstract

*Corydalis humosa* Migo is a traditional Chinese medicine that clears away damp heat, relieves sore. Protopine (PRO) is an alkaloid component isolated from *C. humosa* Migo. However, the role of protopine in acute kidney injury (AKI) has not yet been reported. This study aims to investigate the effect and mechanism of protopine isolated from *C. humosa* Migo on lipopolysaccharide (LPS)-induced AKI in mice. Inflammation accumulation was assessed by small animal living imaging. The blood urea nitrogen (BUN), and serum creatinine (Scr) were measured to assess the effects of protopine on renal function in LPS-induced AKI. The levels of tumor necrosis factor (TNF), interleukin-2 (IL-2), interferon-γ (IFN-γ), and (interleukin-10) IL-10 in serum were detected by cytometric bead array. Flow cytometry was used to detect the levels of reactive oxygen species (ROS) in primary kidney cells. The proportions of granulocytes, neutrophils, and macrophages in peripheral blood were examined to evaluate the effect of protopine on immune cells in mice with AKI. Toll-like receptor (TLR4) and apoptotic signaling pathway were detected by Western blot analysis. The results showed that protopine markedly improved the renal function, relieve inflammation, reversed inflammatory cytokines, transformed apoptosis markers, and regulated the TLR4 signaling pathway in mice with AKI induced by LPS. The protopine isolated from *C.* *humosa* Migo protected mice against LPS-induced AKI by inhibiting apoptosis and inflammation via the TLR4 signaling pathway, thus providing a molecular basis for a novel medical treatment of AKI.

## 1. Introduction

The essence of sepsis is systemic inflammatory reactions. Multiple inflammatory mediators and enzymes participate in organism damage; however, the kidney is the most sensitive organ in sepsis infection [1]. Acute kidney injury (AKI) is a clinical syndrome caused by a variety of pathogens, leading to morbidity and mortality in patients [2]. The pathogenesis is complicated, including pro-inflammatory cytokines, ROS, and cell apoptosis [3,4,5]. Lipopolysaccharide (LPS)-induced sepsis remains the leading cause of AKI. One of the mechanisms of sepsis-induced AKI involves the release of bacterial endotoxins into the circulation, which activate interconnected inflammatory cascades in the kidney, ultimately leading to renal injury [6]. Because of the early excessive inflammatory response, T cells, B cells, and dendritic cells (DC) are severely depleted, and the body’s immune system is in a paralyzed state. An increasing number of studies suggest that immunosuppression in the late stage of sepsis is the main cause of the high mortality rate in patients with sepsis [7].

Pro-inflammatory cytokines have been shown to exacerbate ROS generation [8], which can activate several intracellular signaling pathways including the one that involves the transcription factor NF-κB [9]. ROS is a highly biologically active oxygen molecule, and increased levels of ROS can induce apoptosis [10]. During the development of AKI, a large number of renal tubular epithelial cells in the nephrons are apoptotic, leading to cell necrosis. AKI could lead to the destruction of the mitochondrial outer membrane, resulting in high expression of TNF-α, Bax, and caspase that induce apoptosis. In addition, the expression of an important anti-apoptotic gene *Bcl-2* decreases, inducing apoptosis [11]. Studies have found that LPS can promote the expression of TLR4 and NF-κB apoptosis-related receptors, inducing systemic inflammatory response and causing apoptosis and multiple organ damage [12]. Moreover, TLR4 activation in primary human lung cancer cells increased their ROS levels [13].

*C. humosa* Migo is from the Papaveraceae family, and the tuber of *C. humosa* Migo is used in traditional Chinese medicine, which could clear away damp heat, relieve sore, promote blood circulation, and relieve pain. Studies have shown that the compositions of *C. humosa* Migo tuber extract are mostly alkaloids, such as *l*-tetrahydrocoptisine, hydroprotopine, *dl*-tetrahydrocoptisine, berberine, and *dl*-tetrahydroberberine etc. [14,15]. In our study, protopine is an alkaloid component isolated from *C. humosa* Migo *humosa* Migo and studies have shown that protopine has many demonstrated biological activities, such as anti-inflammatory [16], anti-parasitic [17], antimicrobial [18], and hepatoprotective effects, in animal models [19]. However, the role of protopine in AKI remains unknown. This study was performed to investigate the effects of protopine on LPS-induced AKI and explore the underlying mechanisms.

## 2. Results

### 2.1. Compound Identification and Structural Elucidation

**Figure 1:**
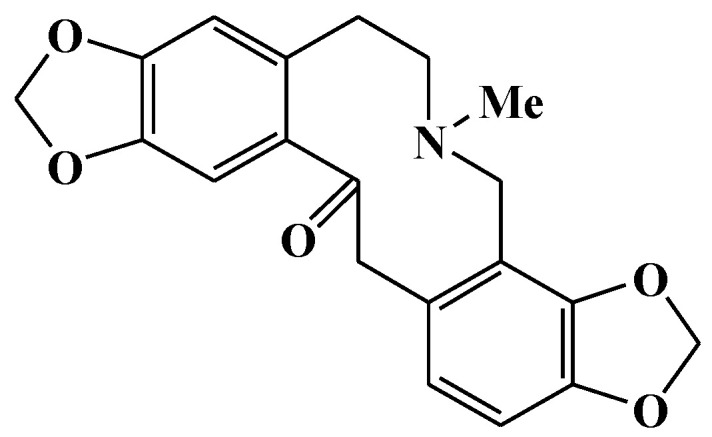
Structure of compound **LDD-16**.

Compound **LDD-16** was obtained as white spheroidal crystals soluble in chloroform. The ferric chloride-potassium ferricyanide reagent does not develop color and the color reaction of the modified iodinated potassium reagent is positive, suggesting that it is an alkaloid component. For the ^1^H-NMR spectrum of 1 (Table 1), eight hydrogen proton signal peaks were observed in the aromatic region; δ 6.94 (1H, s, H-1) and 6.79 (1H, s, H-4) denoted a set of para-hydrogen signals on the benzene ring; δ 6.70 (1H, d, *J* = 8.0 Hz, H-11) and 6.66 (1H, d, *J* = 8.0 Hz, H-12) denoted a set of ortho-hydrogen signals on the benzene ring; δ 5.98 (2H, s) and 5.95 (2H, s) denoted the characteristic signal peak of two methylenedioxy groups. In addition to the hydrogen signal on the *N*-methyl group at δ1.81, eight hydrogen proton signal peaks were observed in the high-field region. The ^13^C-NMR spectrum displayed a total of 20 carbons. Besides a carbonyl group at δ194.6, two methylenedioxy groups were found at δ100.6 and δ101.0. Further, 12 carbon signals were observed, suggesting the presence of 2 benzene rings; δ41.0 was a characteristic signal peak of the methyl group. In the 1H detected heteronuclear multiple bond correlation (HMBC) spectrum, δ6.79 had a cross peak with δ30.4 (C-5) and δ6.66 had a cross peak with δ46.6 (C-13), suggesting that H-4 was adjacent to C-5 and H-12 was adjacent to C-13; δ6.94 had a cross peak with δ194.6 (C-14), suggesting that H-1 was adjacent to C-14; δ5.95 had a cross peak with δ145.7 (C-9) and δ145.1 (C-10), suggesting that methylenedioxy was attached to C-9 and C-10; δ5.98 had a cross peak with δ147.2 (C-2) and 145.2 (C-3), suggesting that methylenedioxy was attached to C-2 and C-3; δ1.81 had a cross peak with δ50.6 (C-8) and 57.3 (C-6), suggesting that both C-6 and C-8 were connected to N. Based on the aforementioned analysis and literature review, the structure of the compound **LDD-16** was determined to be protopine, which is shown in Figure 1.

### 2.2. Effects of Protopine on the Renal Function in Mice with LPS-Induced AKI

**Figure 2:**
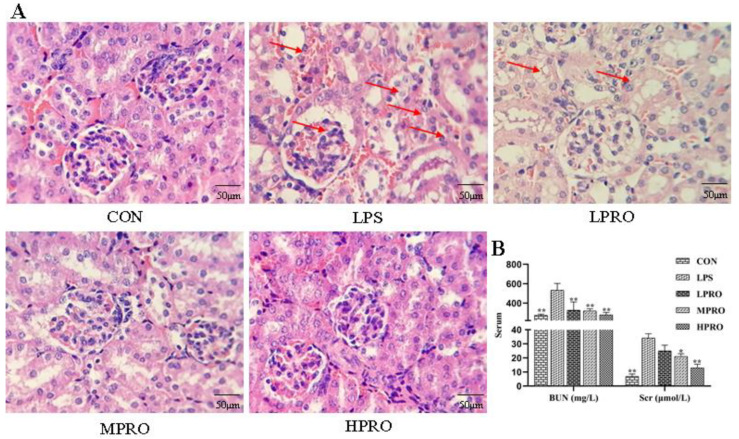
Effects of protopine on the renal function in mice with LPS-induced acute kidney injury (AKI). (**A**) Representative histopathological images for the H&E staining of formalin-fixed kidney tissues from each group (400× magnification); scale bar 50 μm. (**B**) Effects of protopine on Scr and BUN in mice with LPS-induced AKI (*n* = 8 mice per group). * *p* < 0.05, ** *p* < 0.01 compared with the LPS group. (LPRO: low-dose protopine; MPRO: medium-dose protopine; HPRO: high-dose protopine; LPS: lipopolysaccharide; AKI: acute kidney injury; Scr: serum creatinine; BUN: blood urea nitrogen; H&E: hematoxylin and eosin).

As shown in Figure 2, no significant pathological changes were found in the kidneys of control mice. In contrast, obvious renal pathological damage was observed in mice with LPS-induced AKI, including glomerular atrophy, large number of red blood cells, tubular cell vacuolation, tubular dilation and distortion, tubular cell necrosis, and nuclear loss. However, these LPS-induced pathological changes were significantly attenuated by PRO dose-dependently. In addition, the levels of BUN and Scr in mice treated with LPS increased significantly, which were reduced by PRO.

### 2.3. Effects of Protopine on Apoptosis in Mice with LPS-Induced AKI

**Figure 3:**
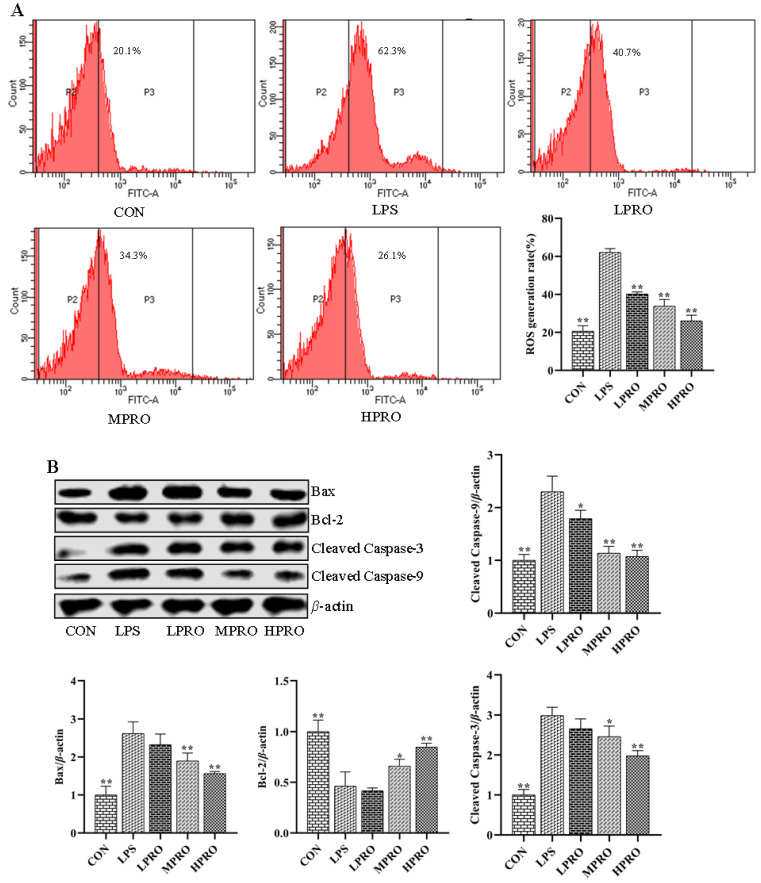
Effects of protopine on cell apoptosis in mice with LPS-induced AKI (*n* = 8 mice per group). (**A**) Effects of protopine on oxidative stress in mice with LPS-induced AKI. (**B**) Effects of protopine on apoptosis marker proteins in mice with LPS-induced AKI. * *p* < 0.05, ** *p* < 0.01 compared with the LPS group. (LPRO: low-dose protopine; MPRO: medium-dose protopine; HPRO: high-dose protopine; LPS: lipopolysaccharide; AKI: acute kidney injury; ROS: reactive oxygen species; FCM: flow cytometry; Bax: associated X protein; Bcl-2: B-cell lymphoma 2).

The effects of protopine on oxidative stress in mice with AKI were investigated by measuring the levels of ROS in primary renal cells using FCM. As shown in Figure 3, the results revealed that the levels of ROS increased in mice with LPS-induced AKI, which could be reduced by protopine.

Increased levels of ROS in the cells could induce apoptosis. The levels of apoptosis marker proteins were determined by Western blot analysis to assess the effect of protopine on LPS-induced apoptosis. As shown in Figure 3, the results demonstrated the downregulation of pro-apoptotic protein Bax, apoptotic promoter caspase-3, and apoptosis executor caspase-9 and the upregulation of Bcl-2 by protopine.

### 2.4. Effects of Protopine on Inflammation in Mice with LPS-Induced AKI

**Figure 4:**
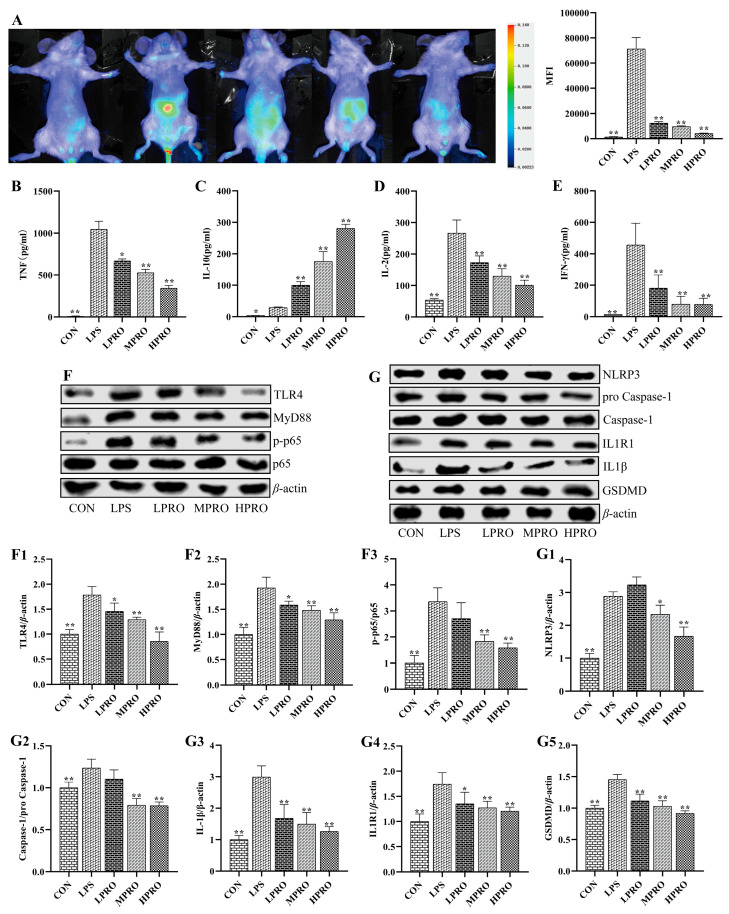
Effects of protopine on inflammatory response in mice with LPS-induced AKI (*n* = 8 mice per group). (**A**) Effects of protopine on inflammation accumulation in mice with LPS-induced AKI detected by small animal imaging. (**B**–**E**) Effects of protopine on inflammatory cytokines in mice with LPS-induced AKI. (**F**,**G**) Effects of protopine on TLR4 signaling pathway in mice with LPS-induced AKI. (**F1**) TLR4. (**F2**) Myd88. (**F3**) p-p65/p65. (**G1**) NLRP3. (**G2**) Caspase1/pro Caspase1. (**G3**) IL-1β. (**G4**) IL1R1. (**G5**) GSDMD. * *p* < 0.05, ** *p* < 0.01 compared with the LPS group. (LPRO: low-dose protopine; MPRO: medium-dose protopine; HPRO: high-dose protopine; LPS: lipopolysaccharide; AKI: acute kidney injury; TLR4: toll-like receptor 4; NLRP3: nod receptor heat protein domain associated protein 3; IL-1β: interleukin-1β; IL1R1: interleukin-1 receptor 1; GSDMD: gasdermin; MyD88: myeloid differentiation factor88).

To assess the effect of protopine on inflammatory response in vivo, inflammation accumulation and some representative inflammatory cytokines were determined by small animal imaging and CBA, respectively. As shown in Figure 4, there is more severe inflammation accumulation in LPS-induced mice compared with control mice, which could be alleviated by protopine. In addition, the levels of IFN-γ, TNF, and IL-2 were significantly increased, which could be reversed by protopine effectively. The level of IL-10 significantly increased in the serum of mice with LPS-induced AKI. However, the level of IL-10 further significantly increased after treatment with protopine.

The effect of protopine on LPS-induced inflammatory response was further explored by examining the levels of a number of proteins related to inflammation. As shown in Figure 4, protopine significantly inhibited the activation of the TLR4/MyD88/NF-κB pathway and reduced the levels of NLRP3, GSDMD, caspase-1/pro caspase-1, and IL1R1 in the kidney of mice with AKI.

### 2.5. Effects of Protopine on the White Blood Cell Subtype in Peripheral Blood of Mice with LPS-Induced AKI

**Figure 5:**
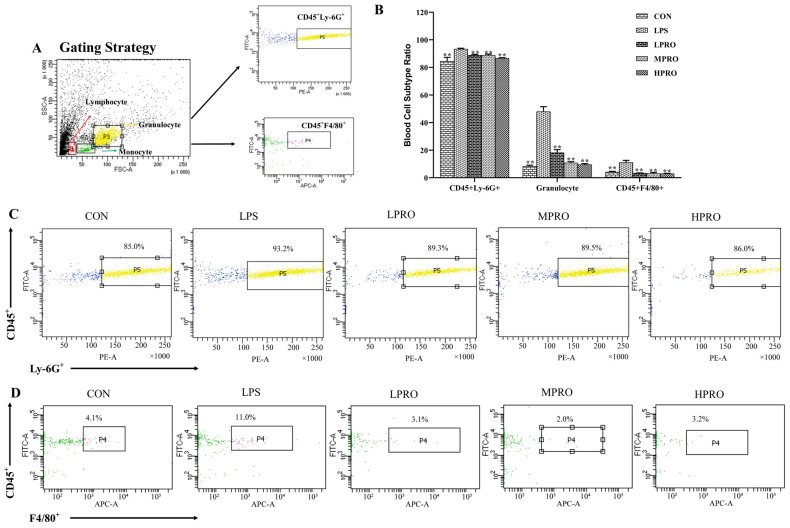
Effects of protopine on the blood cell subtype in mice with. LPS-induced AKI (*n* = 8 mice per group). (**A**) Gating Strategy. (**B**) Effects of protopine on the blood cell subtype ratio in mice with LPS-induced AKI. (**C**,**D**) Effects of protopine on the ratio of CD45^+^Ly-6G^+^ and CD45^+^F4/80^+^ in mice with LPS-induced AKI. ** *p* < 0.01 compared with the LPS group. (LPRO: low-dose protopine; MPRO: medium-dose protopine; HPRO: high-dose protopine; LPS: lipopolysaccharide; AKI: acute kidney injury).

The effects of protopine on the numbers of neutrophils and macrophages in peripheral blood were examined. The results (Figure 5) showed that the numbers of granulocytes, neutrophils, and macrophages significantly increased in peripheral blood of mice with LPS-induced AKI, which could be reversed by protopine effectively.

## 3. Discussion

AKI is a grievous complication followed by sepsis, which could lead to high mortality [3]. A number of studies about AKI were focused on pathophysiological mechanisms and risk factors. However, the therapy of AKI and the causing mortality is still a challenging trouble in clinical practice [3]. *C. humosa* Migo is a traditional Chinese medicine that clears away damp heat, relieves sores, promotes blood circulation, and relieves pain. protopine is a compound isolated from *C. humosa* Migo, which has been reported to have various biological and pharmacological activities; however, the effect of protopine on AKI is not fully understood and needs to be further explored. This study found that protopine has a good protective effect on LPS-induced acute kidney injury in mice for the first time, and explored its possible mechanism of action, which could provide some new information about the use of protopine for treating AKI.

AKI is one of the complications of sepsis, and AKI is followed by a rapid decline in renal function [20]. The renal functions of all groups were evaluated by assessing the levels of BUN and Scr and observing renal histopathological sections stained with H&E in mice with LPS-induced AKI. The mice injected with 5 mg/kg LPS suffered from severe renal pathological damage, including glomerular atrophy, tubular cell vacuolation, tubular dilation and distortion, tubular cell necrosis, and nuclear loss, accompanied by increase in the levels of Scr and BUN. However, these LPS-induced pathological changes were significantly attenuated by protopine, which could also reduce the levels of Scr and BUN in mice with AKI. Therefore, it was preliminarily concluded that protopine had a protective effect on the renal function of animals with LPS-induced AKI.

As we all know, LPS is a crucial exogenous mediator of sepsis, which could give rise to metabolic disorder and organ failure by generating ROS [21] and mitochondria dysfunction. The short-dated outburst of ROS could lead to increased mitochondrial membrane permeability, reduced membrane potential, and release of apoptosis-inducing factors to give impetus to apoptosis. The production of ROS leads to changes in the expression of Bax and Bcl-2 and the activation of caspase-3 and caspase-9. Apoptosis has recently been defined as a critical role of cell death, and studied suggest that the suppression of cell apoptosis could elevate survival in animal models of sepsis [22]. Moreover, our present study confirmed the inhibition effect of protopine on apoptosis induced by LPS. The results of this study showed that protopine could decrease the level of ROS in primary renal cells of mice with AKI, upregulate the level of Bcl-2, and downregulate the levels of Bax, cleaved caspase-3, and cleaved caspase-9. Our study indicates that protopine could inhibit LPS-induced apoptosis.

Studies have shown that sepsis induced AKI is accompanied by destruction of the immune system [23], and unconscionable apoptosis of immune cells can exacerbate sepsis and increase the mortality rate of patients. A grave inflammatory response induced by LPS stimulates neutrophils to emancipate numerous of pro-inflammatory cytokines, which have been reported to play pivotal roles in the pathogenesis of AKI [24]. In AKI, multiple inflammatory mediators are involved in organ damage. Pro-inflammatory cytokines, such as TNF-*α*, IFN-γ, and caspase-1 have been shown to accelerate the progression of LPS-induced AKI [25]. Moreover, the present study indicated that the numbers of granulocytes, neutrophils, and macrophages in peripheral blood significantly increased in mice with LPS-induced AKI, which could be reduced by protopine effectively. In addition, protopine could mitigate inflammation accumulation in mice induced by LPS. The levels of TNF, IFN-γ, and IL-2 elevated significantly in serum in LPS-induced mice, which could be reversed by protopine. Interestingly, the results showed that LPS increased the low levels of IL-10 in control mice. When the body is infected, such as AKI, the level of anti-inflammatory factor IL-10 in serum increases, but it is not enough to resist the injury. protopine could significantly increase the level of anti-inflammatory factor IL-10 in the serum of mice with AKI, thereby enhancing the body’s ability to resist damage.

Toll-like receptor 4 (TLR4) is a classical pathogen recognition receptor of LPS, which plays a critical role in the innate immune system [6,26]. Upon stimulation with LPS, TLR4 can activate myeloid differentiation factor 88 (MyD88) and nuclear factor kappa-B (NF-κB) [27]. TLR4 is activated by pathogen-associated factors, which could activate the NF-κB signaling pathway and sponsor the generation of pro-inflammatory cytokines and chemoattractants [28,29]. The transcription factor NF-κB is a vital element in the inflammatory response, the activated NF-κB is transferred to the nucleus and induce the transcription of target genes, including pro-inflammatory cytokines, in tubular cells and infiltrated immune cells [30]. However, the emancipated inflammatory factor can also activate NF-κB pathway, forming a malicious cycle [31]. Moreover, the present study showed that the levels of TLR4, MyD88, and p-p65 significantly increased in mice induced by LPS, which were reduced by protopine.

Certain microbial components or some endogenous molecules including IL-1β and TNF-α can intervene NLRP3 deliverance through NF-κB p65 produced by TLRs. NLRP3, as a type of inflammasome, which has been reported to activate caspase-1, resulting in the generation of IL-1β [32]. Studies showed that NLRP3 inflammasome accelerate renal inflammatory response by generating inflammatory cytokines and NLRP3 inflammasome knockout mice were protected against kidney injury [33]. After the activation of NLRP3, proteins were oligomerized and tricked through homotypic molecular interactions, adaptor protein apoptosis-associated speck-like protein containing a caspase recruitment domain (ASC), and protease caspase-1 to form the inflammasome [34]. The formation of the inflammasome could lead to the activation of caspase-1, causing the processing of cellular substrates, including the cytokines pro-IL-1β and pro-IL-18 [35]. Interleukin-1 receptor (IL1R) is the receptor of IL-1α and IL-1β. As we all know, the proliferation and differentiation of immune cells, accelerated by IL-1β, causes chemotaxis and agglutination to inflammatory cells, and precipitates the importation of activated immune cells into inflammatory sites. Caspase-1 is the first discovered IL-1β-converting enzyme, which is known as a primary player in inflammation and cell death. Caspase-1 is activated in NLRP3, which is a multiprotein oligomer composed of a certain intracellular pattern recognition receptor, adaptor ASC, and pro-caspase-1 [36]. Recently, gasdermin D (GSDMD) was identified as a determinant mediator of pyroptosis. Active caspase-1, caspase-4, caspase-5, and caspase-11 cleave GSDMD within a linker between its N-terminal and C-terminal domains [37,38]. In addition, GSDMD could induce the release of IL-1β. The present study found that the levels of NLRP3, caspase-1/pro-caspase-1, IL1R1, IL-1β, and GSDMD increased in kidneys of mice with AKI, which could be reduced by protopine. Our study indicates that protopine could inhibit LPS-induced inflammatory response and activation of the TLR4 signaling pathway.

## 4. Materials and Methods

### 4.1. General Experimental Procedure

Nuclear magnetic resonance (NMR) spectroscopy was performed at room temperature using a Bruker Avance III 500 MHz spectrometer with tetramethylsilane (TMS) as a standard. P-HPLC was acquired on the YMC-Pack ODS-A column (250 × 10 mm^2^ and 5 μm) using Saipuruisi LC-50 instrument with a UV200 detector. CC was performed on Diaion HP-20 absorbent (Mitsubishi Chemical Co. Tokyo, Japan) and alumina column, and TLC was performed on custom silica gel G plates (Qingdao Marine Chemical Industry, Qingdao, China). The chemical reagents used for isolation were of analytical grade, and the solvents used for p-HPLC were of chromatographic grade.

### 4.2. Plant Materials

The tubers of *C. humosa* Migo were collected from the Xinzheng ancestral mountain in Henan Province, China. The plant material was identified by Professor Suiqing Chen and Chengming Dong (Henan University of Chinese Medicine). The voucher number (No.20171026) of *C. humosa* Migo was deposited in the Research Department of Natural Medicinal Chemistry, School of Pharmacy, Henan University of Chinese Medicine.

### 4.3. Extraction and Isolation

The tubers of *C. humosa* Migo. (35 kg) were extracted by tissue fragmentation method with 70% acetone three times, concentrated and evaporated, and then dissolved in water with water-soluble substances (320 g). The water-soluble substances were resolved on a Diaion HP-20 macroporous resin column and successively eluted with MeOH-H2O (0:100, 20:80, and 40:60) to obtain three fractions (F1–F3). F3 (37.2 g) was suspended in MeOH and chromatographed on an alumina column eluted with CH2Cl2-MeOH (80:1–1:1). The alumina column was purified by repeated column chromatography and combined with preparative liquid-phase recrystallization method to obtain the compound named **LDD-16** (20 mg).

### 4.4. LPS-Induced AKI In Vivo

The study was conducted in accordance with the regulations for experimental animal management promulgated by the National Science and Technology Commission of the People’s Republic of China. In this experiment (the license number for animal experiment: SYXK2015-0005), male BALB/c mice (20 ± 2 g, *n* = 75) were purchased from Beijing Vital River Laboratory Animal Technology Co., Ltd. (SCXK2016-0006). They were adapted to the environment for a week and provided regular food and water every day. The mice were randomly divided into five groups (*n* = 15 per group) as follows: control (CON), LPS (5 mg/kg), LPRO + LPS (low-dose protopine, 10 mg/kg), MPRO + LPS (medium-dose protopine20 mg/kg), and HPRO + LPS (high-dose protopine 30 mg/kg). All mice were injected intraperitoneally with LPS, except those in the normal group that were injected intraperitoneally with saline. After 3 h, different doses of protopine were administered intragastrically. The CON and LPS were given distilled water at the same time by gavage twice a day for 3 days. After 1 h of the last administration, inflammation accumulation was assessed by small animal living imaging. And after 24 h of the last administration, blood was extracted from their eyeballs, and the kidneys were carefully removed immediately and stored in the liquid nitrogen. The blood was centrifuged at 3000 rpm for 10 min to obtain the supernatant. The blood and the kidney samples were stored at −80 °C for further analysis.

### 4.5. Flow Cytometry Analysis of White Blood Cell Subtype

Whole blood anticoagulated with heparin sodium (100 μL) was conjugated with fluoresceine isothiocyanate (FITC) rat anti-Mouse CD45 (553080, BD, USA), phycoerythrin (PE) rat anti-mouse Ly-6G and Ly-6C (553128), and Alexa Fluor 647 rat anti-mouse F4/80 (565853, BD) at room temperature for 30 min. Then, 2 mL of 1× red cell lysate was added for 10 min at room temperature in the dark and centrifuged for 5 min at 300× *g*. The supernatant was discarded, and 2 mL of phosphate-buffered saline (PBS) was added for resuspension and centrifuged at 300× *g* for 5 min. The supernatant was discarded again, and 500 μL of PBS was added, followed by analysis by flow cytometry (FCM; FACS Aria III, San Jose, CA, USA).

### 4.6. Primary Kidney Cells and Analysis of ROS

Fresh kidney tissue (10 mg) was taken, chopped to a size of about 0.5 mm^3^, washed twice with PBS, and then resuspended in 0.25% trypsin digestion. Fetal bovine serum was used to stop digestion. The tissue was rinsed with PBS, filtered with 70-μm cell sieve, and centrifuged to obtain primary kidney cells. The resulting cells were used to detect the level of ROS by FCM using the commercially available kit (CA1410, Beijing Solarbio Science &Technology Co., Ltd., Beijing, China) according to the manufacturer’s recommendation. Briefly, the cells were collected and incubated with 10 μM 2′,7′-dichlorofuorescein (DCFH-DA) for 20 min at 37 °C in the dark. At the end of the incubation, the cells were washed with PBS three times to remove the free DCFH-DA molecules. All assays were performed in triplicate.

### 4.7. Cytometric Bead Array Assay

The contents of IL-10 (Mouse IL-10 Flex Set, 558300, BD), IFN-γ (Mouse IFN-γ Flex Set, 558296, BD), TNF (Mouse TNF Flex Set, 558299, BD), and IL-2 (Mouse IL-2 Flex Set, 558297, BD) in the serum of mice were detected using CBA according to the manufacturer’s protocols. Initially, the different concentrations of standard products were formulated according to the respective preparations, and a standard curve was plotted. Then, 50 μL of serum and 50 μL of microspheres were mixed and incubated at room temperature in the dark for 2 h. Then, 50 μL of PE antibody conjugated with TNF, IL-2, IL-1β, and IFN-γ was added and incubated for 1 h at room temperature in the dark. Further, 1 mL of washing solution was added and centrifuged, and the supernatant was gently discarded. The resulting suspension was resuspended in 400 μL of the buffer, and finally, the cytokine levels were measured by FCM, and the results were analyzed using the Diva (BD).

### 4.8. Assessments of Biochemical Parameters

The levels of BUN (C013-2, Nanjing Jiancheng Bioengineering Institute, Nanjing, Jiangsu, China) and Scr (C011-2, Nanjing Jiancheng Bioengineering Institute) in the serum of mice were detected using specific kits according to the manufacturer’s instructions. All assays were performed in triplicate.

### 4.9. Histomorphological Examination

The kidneys were fixed in freshly prepared 4% paraformaldehyde and embedded. Each paraffin-embedded sample was cut into 5-μm-thick sections and stained with hematoxylin and eosin. The images were viewed and acquired using a microscope (Nikon, Tokyo, Japan).

### 4.10. Western Blot Analysis

Total protein was extracted from the renal tissue according to the instructions of the mammalian protein extraction kit (Beijing Com Win Biotech Co., Ltd., Beijing, China) and quantified using the Bradford protein assay kit (Wuhan Boster Biological Technology., Ltd., Wuhan, Hubei, China). Protein (60 μg) was loaded per sample and separated using the SDS-PAGE gel. Then, non-fat milk was used to block the membrane for 1.5 h, and the following primary antibodies were added: Bcl-2 (ab59348, Abcam, MA, USA), Bax (ab59348, Abcam), Caspase-3 (ab13847, Abcam), Caspase-9 (ab32539, Abcam), NF-κB p65 (ab16502, Abcam), NF-κB p-p65 (ab56299, Abcam), TLR4 (ab22048, Abcam), NLRP3 (ab4207, Abcam), IL1R1 (ab106278, Abcam), IL-1β (ab9722, Abcam), GSDMD (ab209845, AbcamS), MyD88 (ab135693, Abcam), Caspase-1 (ab1872, Abcam), pro-Caspase-1 + p10 + p12 (ab179515, Abcam), and β-actin (ab8227, Abcam), incubated overnight at 4 °C, and washed five times with PBST for 5 min each. Then, secondary antibodies (goat anti-rabbit 925-68071 and goat anti-mouse 925-32210, Li-COR, MO, USA) were added, incubated for 1 h in the dark, and washed four times with PBST for 5 min each time and finally with PBS. The expression of the proteins was quantified by Odyssey (CLx, Li-COR, Biosciences, Lincoln, NE, USA).

### 4.11. Statistical Analysis

Data were analyzed using the SPSS 20.0 software (IBM; Armonk, NY, USA). Statistical significance was assessed in comparison with the respective control for each experiment using one-way analysis of variance. A *p* value less than 0.05 indicated a statistically significant difference.

## 5. Conclusions

In summary, the present study for the first time indicated that protopine isolated from *C. humosa* Migo protected mice against LPS-induced AKI by inhibiting apoptosis and inflammation, which might be related to the inhibition of TLR4 signaling pathway after the mice treated with protopine, thus providing a molecular basis for a novel medical treatment of AKI.

## Figures and Tables

**Table 1:** NMR data for compound **LDD-16** in DMSO-*d_6._*

NO.	*δ*_H_	*δ*_C_
1a		132.6
1	6.94 (1H, s)	107.2
2		147.2
3		145.2
4	6.79 (1H, s)	106.2
4a		135.9
5		30.4
6		57.3
8		50.6
8a		118.2
9		145.7
10		145.1
11	6.70 (1H, d, *J* = 8.0 Hz)	110.3
12	6.66 (1H, d, *J* = 8.0 Hz)	124.9
12a		129.5
13		46.0
14		194.6
-OCH_2_O-	5.95 (2H, s)	100.6
	5.98 (2H, s)	101.0
N-CH_3_	1.81 (3H, s)	41.0

## Abbreviations

*C. humosa* Migo	*Corydalis humosa* Migo
PRO	protopine
LPS	lipopolysaccharide
AKI	acute kidney injury
BUN	blood urea nitrogen
Scr	serum creatinine
TNF	tumor necrosis factor
IL-2	interleukin-2
IFN-γ	interferon-γ
IL-10	interleukin-10
ROS	reactive oxygen species
TLR4	toll-like receptor 4
NLRP3	nod receptor heat protein domain associated protein 3
NMR	nuclear magnetic resonance
TMS	tetramethylsilane
DMSO	dimethyl sulfoxide
FITC	fluoresceine isothiocyanate
H&E	hematoxylin and eosin
Bax	associated X protein
Bcl-2	B-cell lymphoma 2
CBA	cytometric bead array
IL-1β	interleukin-1β
IL1R1	interleukin-1 receptor 1
GSDMD	gasdermin
MyD88	myeloiddifferentiationfactor88
