# Correction: Zhang et al. Protopine Protects Mice Against LPS-Induced Acute Kidney Injury by Inhibiting Apoptosis and Inflammation via the TLR4 Signaling Pathway. *Molecules* 2020, *25*, 15

**DOI:** 10.3390/molecules31050806

**Published:** 2026-02-28

**Authors:** Beibei Zhang, Mengnan Zeng, Meng Li, Yuxuan Kan, Benke Li, Ruiqi Xu, Yuanyuan Wu, Shengchao Wang, Xiaoke Zheng, Weisheng Feng

**Affiliations:** 1 College of Pharmacy, Henan University of Chinese Medicine, Zhengzhou 450046, China; zhangs9426@163.com (B.Z.); 17320138484@163.com (M.Z.); limeng31716@163.com (M.L.); kanyx0827@163.com (Y.K.); libk2017@163.com (B.L.); xuruiqi9647@163.com (R.X.); wyy96191711@163.com (Y.W.); wangsc1204@163.com (S.W.); 2 Collaborative Innovation Center for Respiratory Disease Diagnosis and Treatment & Chinese Medicine Development of Henan Province, Zhengzhou 450046, China

## Error in Figure

In the original publication [1], there was a mistake in Figure 4G and Figure 5 as published. The bonds of IL1β in Figure 4 were copied by mistake, and the corrected Figure 4 appears below. In addition, Figure 5C was also copied by mistake, and the corrected Figure 5 appears below. The authors state that the scientific conclusions are unaffected. This correction was approved by the Academic Editor. The original publication has also been updated.

## Figures and Tables

**Figure 4:**
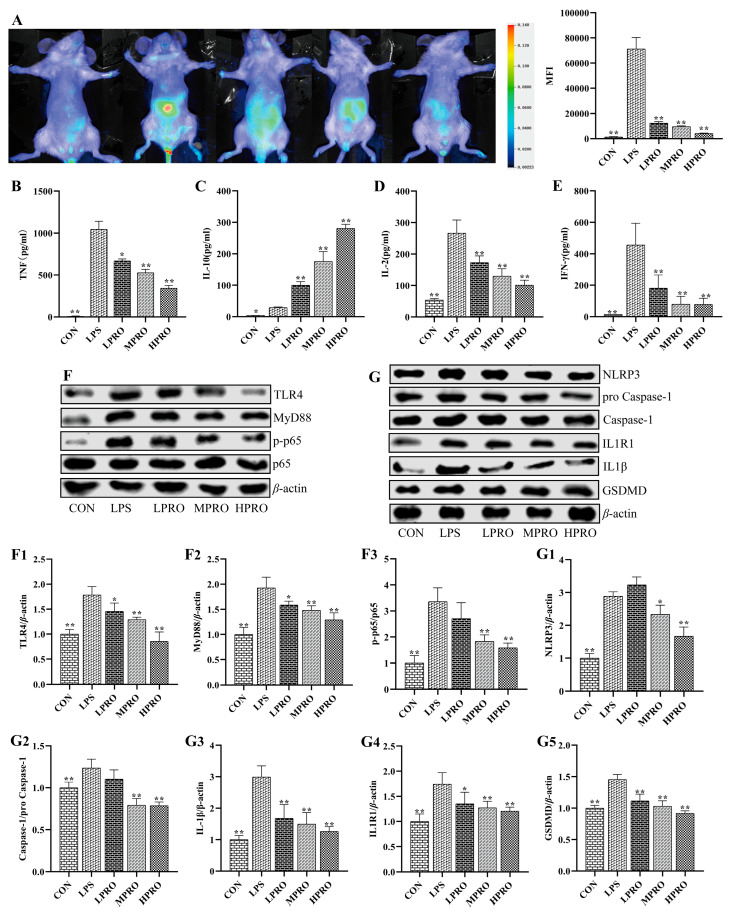
Effects of protopine on inflammatory response in mice with LPS-induced AKI (*n* = 8 mice per group). (**A**) Effects of protopine on inflammation accumulation in mice with LPS-induced AKI detected by small animal imaging. (**B**–**E**) Effects of protopine on inflammatory cytokines in mice with LPS-induced AKI. (**F**,**G**) Effects of protopine on TLR4 signaling pathway in mice with LPS-induced AKI. (**F1**) TLR4. (**F2**) Myd88. (**F3**) p-p65/p65. (**G1**) NLRP3. (**G2**) Caspase1/pro Caspase1. (**G3**) IL-1β. (**G4**) IL1R1. (**G5**) GSDMD. * *p* < 0.05, ** *p* < 0.01 compared with the LPS group. (LPRO: low-dose protopine; MPRO: medium-dose protopine; HPRO: high-dose protopine; LPS: lipopolysaccharide; AKI: acute kidney injury; TLR4: toll-like receptor 4; NLRP3: nod receptor heat protein domain associated protein 3; IL-1β: interleukin-1β; IL1R1: interleukin-1 receptor 1; GSDMD: gasdermin; MyD88: myeloid differentiation factor88).

**Figure 5:**
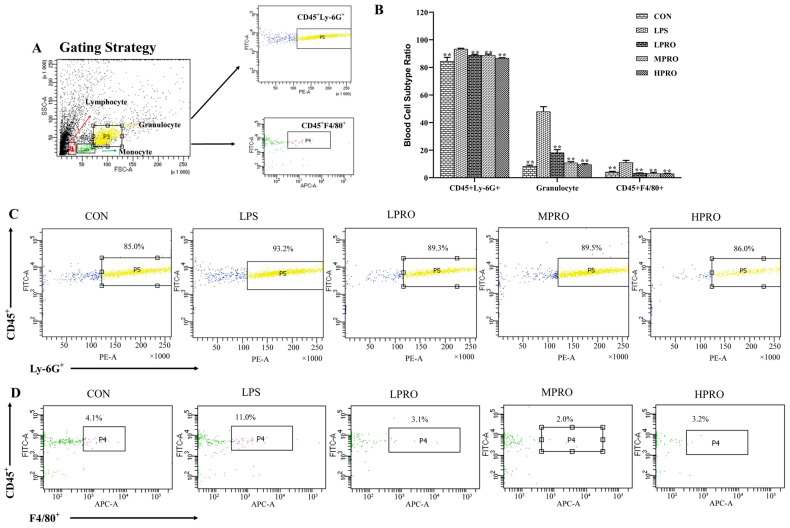
Effects of protopine on the blood cell subtype in mice with. LPS-induced AKI (*n* = 8 mice per group). (**A**) Gating Strategy. (**B**) Effects of protopine on the blood cell subtype ratio in mice with LPS-induced AKI. (**C**,**D**) Effects of protopine on the ratio of CD45^+^Ly-6G^+^ and CD45^+^F4/80^+^ in mice with LPS-induced AKI. ** *p* < 0.01 compared with the LPS group. (LPRO: low-dose protopine; MPRO: medium-dose protopine; HPRO: high-dose protopine; LPS: lipopolysaccharide; AKI: acute kidney injury).
